# Tensions experienced by case managers working in home care for older adults in Quebec: first level analysis of an institutional ethnography

**DOI:** 10.1186/s12913-024-10709-6

**Published:** 2024-03-06

**Authors:** Alexandra Ethier, Marie-France Dubois, Virginie Savaria, Annie Carrier

**Affiliations:** 1 École de réadaptation, Faculté de médecine et des sciences de la santé , Université de Sherbrooke; Centre de recherche sur le vieillissement, Centre intégré universitaire de santé et de services sociaux de l’Estrie - Centre hospitalier universitaire de Sherbrooke, 3001 12e Avenue N, Sherbrooke, QC J1H 5H3 Canada; 2 Département des sciences de la santé communautaire, Faculté de médecine et sciences de la santé, Université de Sherbrooke; Centre de recherche sur le vieillissement, Centre intégré universitaire de santé et de services sociaux de l’Estrie - Centre hospitalier universitaire de Sherbrooke, 3001 12e Avenue N, Sherbrooke, QC J1H 5H3 Canada

**Keywords:** Case management, Older adults, Performance indicators, Discharge planning, Quality, Standardised assessment

## Abstract

**Background:**

Case managers play a vital role in integrating the necessary services to optimise health-related goals and outcomes. Studies suggest that in home care, case managers encounter tensions in their day-to-day work, that is, disjuncture between what they should do, in theory, and what they actually do, in practice. However, direct exploration of these tensions is lacking. As such, this study aimed to describe the tensions encountered by case managers in public home care for older adults in Quebec and their influence on day-to-day work.

**Methods:**

An institutional ethnography was conducted through observations of work, interviews and a survey with case managers working in home care in Quebec. Data were analysed using institutional ethnography first-level analysis procedures. This included mapping the work sequences as well as identifying the tensions experienced by case managers through the words they used.

**Results:**

Three main tensions were identified. First, case managers perceive that, despite working to return hospitalised older adults at home safely, their work also aims to help free up hospital beds. Thus, they often find themselves needing to respond quickly to hospital-related inquiries or expedite requests for home care services. Second, they are supposed to delegate the care to “partners” (e.g., private organisations). However, they feel that they are in effect managing the quality of the services provided by the “partners.” Consequently, they go to great lengths to ensure that good care will be provided. Finally, they must choose between meeting organisational requirements (e.g., reporting statistics about the work, documenting information in the older adults’ file, doing mandatory assessments) and spending time providing direct care. This often leads to prioritising direct care provision over administrative tasks, resulting in minimal reporting of essential information.

**Conclusion:**

The results are discussed using the three lenses of professional practice context analyses (i.e., accountability, ethics, and professional-as-worker) to formulate recommendations for practice and research. They suggest that, despite their important role, case managers have limited power in home care (e.g., with partners, with the hospital).

**Supplementary Information:**

The online version contains supplementary material available at 10.1186/s12913-024-10709-6.

## Background

Performant health and social care systems rely, among other things, on home care services [1]. In Canada, there are no federal standards for home care with which provinces must comply because home care is an extended service [2]. Therefore, each province organises home care, according to their needs. However, most home care programmes across the Canadian provinces seem to share the provision of care and services at the older adults’ home by their informal caregivers and workers (e.g., nurses, social workers, home support workers) [3]. These include, but are not restricted to, nursing, rehabilitation, bathing, feeding, toileting, managing medication, home maintenance [4] which aim to keep older adults at home, as long as it is safe and possible [3].

However, keeping older adults at home safely can be difficult. Indeed, some older adults require extensive help to age in place. In a study conducted in the Canadian province of Quebec, it has been found that in 2019–2020, 10% of home care users received 69% of all interventions provided in home care [5]. This included nursing, professional services, as well as home support. This observation may be attributed to the prevalence of multiple health conditions among some older adults receiving home care [6, 7], leading to a variety of health-related needs [8, 9]. Thus, older adults with multiple health conditions may require the involvement of multiple providers.

In many Canadian provinces, services are provided through a mix of public, private and non-profit organisations [10] and informal caregivers [8]. Dealing with the healthcare needs of older adults can be challenging for healthcare professionals who are part of a multi-provider system [11–14] as well as for the older adults receiving these services [15, 16]. This could explain why studies conducted in home care report unmet needs, including in Canada [9, 17, 18] and Quebec [5]. To address these challenges, a case management model offers a valuable solution. Indeed, case management can be defined as a process to help a home care recipient develop a plan that coordinates and integrates the services needed to optimise their health-related goals and outcomes [19, 20]. As such, it involves assigning a professional to assess and respond to the home care recipient’s needs and their interconnections [21].

Case managers are often professionals extensively trained in clinical, medical, psychosocial, or rehabilitation [22]. Their role is to assess older adults with complex needs as well as plan and coordinate services according to those needs [21], through the development of individualised plans, connections with relevant service providers, problem solving and reassessment [23]. Case managers intervene directly and indirectly with older adults. For example, direct interventions are those done during home care visits, with the older adults and/or their informal caregivers [24]. This can be, for example, an assessment of the older adult’s health situation or the teaching of relevant skills necessary for self-managed care [25]. Indirect interventions are those done for the older adult or the informal caregiver, in their absence. This includes the considerable amount of time they spend filling out paperwork and doing administrative tasks [24, 26, 27]. These tasks are important, as they can avoid unnecessary delays in treatments and duplication in the provision of services [28].

Numerous studies have documented the multifaceted role of case managers. Case managers perceive that it includes facilitation of administrative processes, attention to each older adult’s needs, as well as collection and interpretation of the information about the older adult to other workers [29]. Additional studies presented below shed light on their actual work. When they assess the need for home care services, case managers need to take into account both the needs of the older adults and those of their informal caregivers [8]. Their assessment must be comprehensive, including attention to medical, environmental, financial, legal and social factors [28]. In some organisations, they have to take on the stressful responsibility of overseeing financial allocations for services such as rehabilitation, a responsibility with which they are uncomfortable [30]. Case managers also need to work with different organisations (e.g., healthcare agencies) [29, 31, 32] which can be challenging [27, 32]. For example, case managers face difficulties conciliating the imperatives of different organisations such as last-minute discharge of older adults from acute care [33]. As they work under pressure, they have to choose their priorities and delegate tasks to other people involved, including the informal caregivers [34].

Studies also suggest that case managers seem to encounter tensions in their day-to-day work, that is, disjuncture between what they are supposed to do and what they actually do [35]. For example, they have to resort to “workarounds” to meet clients’ needs [27, 33]. They face confusion about their formal role as opposed to what they do in practice [36, 37]. Case managers also encounter difficulties in communicating with external providers from their organisation, which interferes with and reduces the time they have with older adults [27]. Additionally, they struggle with limited financial resources to meet their clients’ needs [26]. However, the direct exploration of these tensions is lacking, leaving their potential influence on case managers’ work unknown.

Knowing more about the tensions experienced by case managers could be the first step to allowing the optimisation of home care. Indeed, with an aging population, many countries aim to provide efficient care [38, 39] and case managers play a pivotal role in this [33]. By targeting where tensions arise in their work, recommendations could be provided to improve models of case management for older adults [40] and lead to greater work satisfaction for case managers. Consequently, we aimed to describe the tensions encountered by case managers in home care programmes for older adults in Quebec and their influence on day-to-day work.

## Methods

This study is part of a larger sequential exploratory mixed-method study related to the work of case management in home care. In the first phase of the study, we conducted an institutional ethnography [41]. Institutional ethnography is an approach which aims to explicate how people’s lives are socially organised and coordinated [42]. It uses a two-level analysis, which relies on two types of key informants: Standpoint informants and secondary informants. In institutional ethnography, participants are called informants because they possess knowledge about specific practices [41]. The first level analysis starts by taking a standpoint (here of case managers) and looks at the actual work of the people from that standpoint. Work is defined as “[…] anything done by people that takes time and effort, that they mean to do, that is done under definite conditions and with whatever means and tools, and that they may have to think about.” (p. 151) [41]. Once institutional ethnographers have a clear idea about the actual work, they specifically look at the tensions experienced by the standpoint informants [35]. In the second level analysis, one tension is chosen to be studied in more depth, becoming the problematic [43]. Then, institutional ethnographers look at how this problematic is shaped by external forces. To do so, they carefully look at the language used on the day-to-day work (first level analysis) [35] and trace how that language is linked to texts used locally and to “boss texts”. These boss texts are documents positioned at the top of a hierarchy of texts (e.g., laws, regulations, policies, etc.) which will be used to coordinate the work at the local level [42]. Thus, institutional ethnographers talk to secondary informants, they collect the documents used or discussed by the informants and map the work, the texts and the people involved. As done by other institutional ethnographies [e.g., 44–48], this article focuses only on results from the first level of analysis [49]. Indeed, second level analysis will be published elsewhere. These first-level results are useful as they explicate everyday experiences, including the tensions shared among standpoint informants [49]. From these tensions can stem recommendations for public home care providers to improve case managers’ working conditions. In our study, we define home care as an institution in which health and social services in the older adult’s home are offered by various providers. Their goal is to keep older adults at home safely, for as long as possible.

### Context

In the province of Quebec, Health and Social Services are organised in 18 different regional areas. Within each area is found at least one Integrated [University] Health and Social Service Centre (I[U]HSSC). These centres play a crucial role in delivering a diverse array of health and social services through a variety of programmes [50]. Specifically, our study focuses on the provision of home care to older adults within the Support Programme for the Autonomy of Seniors (SAPA; *Soutien à l’autonomie des personnes âgées*). This province-wide initiative operates under the regulatory framework of “At home: The first choice - Home support policy” (*Chez soi le premier choix: la politique de soutien à domicile*) [51] and the Act respecting health and social services (*Loi sur les services de santé et les services sociaux*) [52], which are some of the boss texts in Quebec home care [44, 53].

The SAPA programme aims to compensate for disabilities associated with ageing, diverging from programmes like those catering to individuals with significant and persistent disabilities related to various impairments or degenerative illnesses [54]. Eligibility for SAPA is not solely determined by age but rather by the nature of acquired disabilities. The range of services offered within SAPA encompasses a broad spectrum, spanning professional services such as nursing, occupational therapy, social work, physiotherapy, nutrition, and respiratory therapy, to assistance with essential daily activities like washing, toileting, dressing, feeding, transferring, and respite care, that are paid for by public services. Central to the SAPA programme is the mandatory assessment of older adults via a standardised province-wide assessment tool known as the Outil d’évaluation multiclientèle (OEMC). The extent of service (e.g., number of hours for hygiene per week) is determined following an assessment of needs conducted by the case managers. While public home care services are primarily delivered by workers (e.g., professionals, technicians, home support workers) within the SAPA program, some services are financed by the programme but delivered by collaborating organisations referred to as “partners”. These partners include private organisations such as seniors’ residences, private providers and non-profit organisations such as community organisations and domestic help social economy businesses (*Entreprise d’économie sociale en aide à domicile* (*ÉÉSAD*)). The responsibility for case management is distributed among various professionals, including social workers, nurses, occupational therapists, and technicians in social work or special education. Technicians are health associate professionals [22] who undergo a comprehensive three-year technical training program.

The main tasks of case managers include (1) assessing the needs of older adults and intervening accordingly (e.g., allocating and/or referring to other services within and outside the home care programme), (2) facilitating inpatient admission to hospitals and being involved in managing the discharge process to resume home care, (3) providing psychosocial follow-up to older adults and their informal caregivers (e.g., emotional support, looking for new needs), (4) supporting the older adults’ transition to public long-term care housing when staying at home is deemed unsafe.

Our study was conducted in one of the I[U]HSSC, selected purposively, since it comprises both rural and urban populations. It serves approximately 500,000 people, 25,000 of whom receive at least one type of home care service (e.g., nursing, social work). This I[U]HSSC is subdivided into 9 different local territories, called local services networks. These local services networks organise the provision of SAPA programme home care services at the local level.

### Participants and recruitment

To gain access to the standpoint informants, after obtaining ethical approval from the chosen I[U]HSSC, we sent an email to the managers in the different local service networks to solicit their participation in the study. In total, managers from three local service networks agreed to do so. Thus, our sample of settings was chosen conveniently.

We recruited standpoint informants with the help of their managers, who shared an email about the project, as well as by word-of-mouth. As much as possible, we aimed for our sample to ensure the representativeness of each of the various training and of men, as they are underrepresented in home care. Thus, our sampling strategy for standpoint informants was purposive (Table 1). We established that standpoint informants had to be working in this specific programme for at least 6 months to have sufficient knowledge about day-to-day work, as done in other studies in home care [e.g., 12, 55]. In institutional ethnography, first-level data collection and analysis stop once researchers have a sufficient, in-depth understanding of the work that is done [43]. We established that data collection and analysis would stop once the main tasks were mapped and validated by standpoint informants (see next section).

**Table 1:** Contextual information about the standpoint informants

Number of participating case managers	23
**Gender**	
Men	5
Women	18
**Training**	
Social work^1^	16
Occupational therapist	1
Nursing	4
Other^1^	2
**Years of experience in home care** ^2^	
1 to 4	14
5 to 9	3
10 and more	5

^1^ To preserve the standpoint informants’ anonymity, some categories were grouped, or some trainings were hidden.

^2^ One standpoint informant did not provide their years of experience

### Data collection and analysis

We collected and analysed the data iteratively using the institutional ethnography first-level analysis process [49]. Specifically, data analysis consisted of mapping the work done by case managers and identifying the tensions. Data collection and analysis were performed by the first (A1) and third (A3) authors, who are experienced in qualitative data analysis. To map the work and to identify the tensions, A1 collected data through a sociodemographic questionnaire, observations, obser-views, interviews, and a survey. The number of standpoint informants for each combination of data collection methods is presented in Table 2. All the data collection tools (sociodemographic questionnaire, observation grid, interview guide and survey) were developed for the study.

**Table 2:** Number of case managers according to the data collection methods

Data collection method	Number of key informants
Observations and obser-views	4
Observations, obser-views and survey	2
Observations, obser-views, interviews, and survey	2
Interviews only	1
Survey only	8
Interviews and survey	6
Total number of case managers who took part in the study	23

First, to describe the sample, each standpoint informant answered a self-administered sociodemographic questionnaire (supplementary file). The data collected included gender, years of experience and training and practice settings.

Second, to understand the work done by the case managers, A1 observed their work at their office and during home care visits (*n* = 160 h, 25 different days including some full days, mornings only or afternoons only) from July to December 2022, without interfering. A1 accompanied these observations with obser-views (20.5 h). Obser-views are a data collection method based in co-construction of the data collection and analysis [56]. During obser-views, the researcher’s observation of a participant is immediately followed by a discussion about what was observed. In the context of this study, obser-views allowed to clarify the work observed by directly asking the standpoint informant. To conduct the obser-views, during observations, when appropriate, A1 documented key words of observed situations in an observation grid (supplementary file), which contained the actions, the people present, the texts involved, and questions. Then, when possible, A1 audio-recorded the discussion part of the obser-views. During these, A1 asked the questions noted during observations. This included questions such as “What was that action?,” “Who were you talking to?,” and “What does [enter specific word] mean?” Finally, after each day of data collection, A1 used the key words from the observation grid and transcribed the obser-views to create a narrative text describing the case manager’s day. A1 deleted all identifying information in the narrative text.

For each day of observation, A1 and A3 analysed the data by creating maps of the day of work. These maps captured the actions, accompanying words used by case managers, involved individuals, utilised texts and the tensions. As in other institutional ethnographies in home care [e.g., 27, 44, 57], prior to starting our inquiry, we were aware that case managers encountered tensions in their work. However, we were not aware of what those tensions were. Thus, we looked for instances of tension (e.g., expected vs. actual actions [35, 42, 49]) reported by standpoint informants. We marked them with a symbol alongside the corresponding actions in the map. A1 and A3 paid particular attention to expressions such as “I am supposed to,” “in theory,” “my manager or team leader would like me to,” etc. during the entire data collection process, as well as discrepancies between observed actions and the language used by case managers. With the progression of data collection, the workday maps allowed A1 and A3 to develop task-specific maps (e.g., requesting home care services, getting a new older adult in the caseload).

Once we had a clear idea about the work done by case managers, interviews were conducted from February to May 2023 using an interview grid (supplementary file). Interviews were used to explore further observed aspects and validate the mapped work sequences. Specifically, A1 showed and explained the mapping of the specific tasks to standpoint informants who then had to pinpoint on the maps any instances where they identified missing actions or documents. After each interview, A1 and A3 modified the maps according to the data collected. Eight interviews were necessary to validate task-specific maps, meaning that after the eighth interview, no new information was added [58].

After A1 and A3 completed the maps, A1 wrote a synthesis describing the work, highlighting the main tasks, and describing the common tensions lived by the case managers. The synthesis was then reviewed by the second and last authors to ensure its accuracy, increase its clarity and suggest further aspects to be investigated.

Finally, we conducted a survey inspired by the Delphi consultation method with 19 standpoint informants in June of 2023. Delphi consultation allows gaining a consensus among a group of experts (here, the case managers) regarding a specific subject that will be used prospectively (here, the problematic to explore in the second level analysis) [59]. Using a consensus method appeared sensible as institutional ethnography focuses on what is common across people and contexts [60] and requires to start from the interests and concerns of actual people [43]. The survey was thus used to ensure that the identified tensions were shared among case managers from different training, years of experience or gender, as these aspects have been found to influence health professional practice [61], choice of service [62] or well-being at work [63], which could influence the quality of services provided [64]. The survey was not used to quantify the frequencies of those tensions. Only at this stage were standpoint informants made aware of the tensions that were being studied. To conduct the survey, we presented to the standpoint informants the definition of a tension, namely “a disjuncture between what you ***should do*** and what you ***actually do***”. We also presented them with a list of all the tensions previously identified (n = 7; Table 3) in the mapping. To avoid any primacy effect [65], they were presented randomly to each standpoint informant. Standpoint informants had to rank the occurrence of each tension in day-to-day work as nonapplicable, rarely met, regularly met, or almost always/always met. They could also suggest another tension, but they didn’t. We choose to present tensions that were reported by at least 50% of standpoint informants as regularly met, or almost always/always met. Indeed, studies using the Delphi consultation method report that items below 50% show no consensus at all [66]. Also, the 50% cut-off enables many differing perspectives to be identified and used for a further round [67].

## Results

**Fig. 1:**
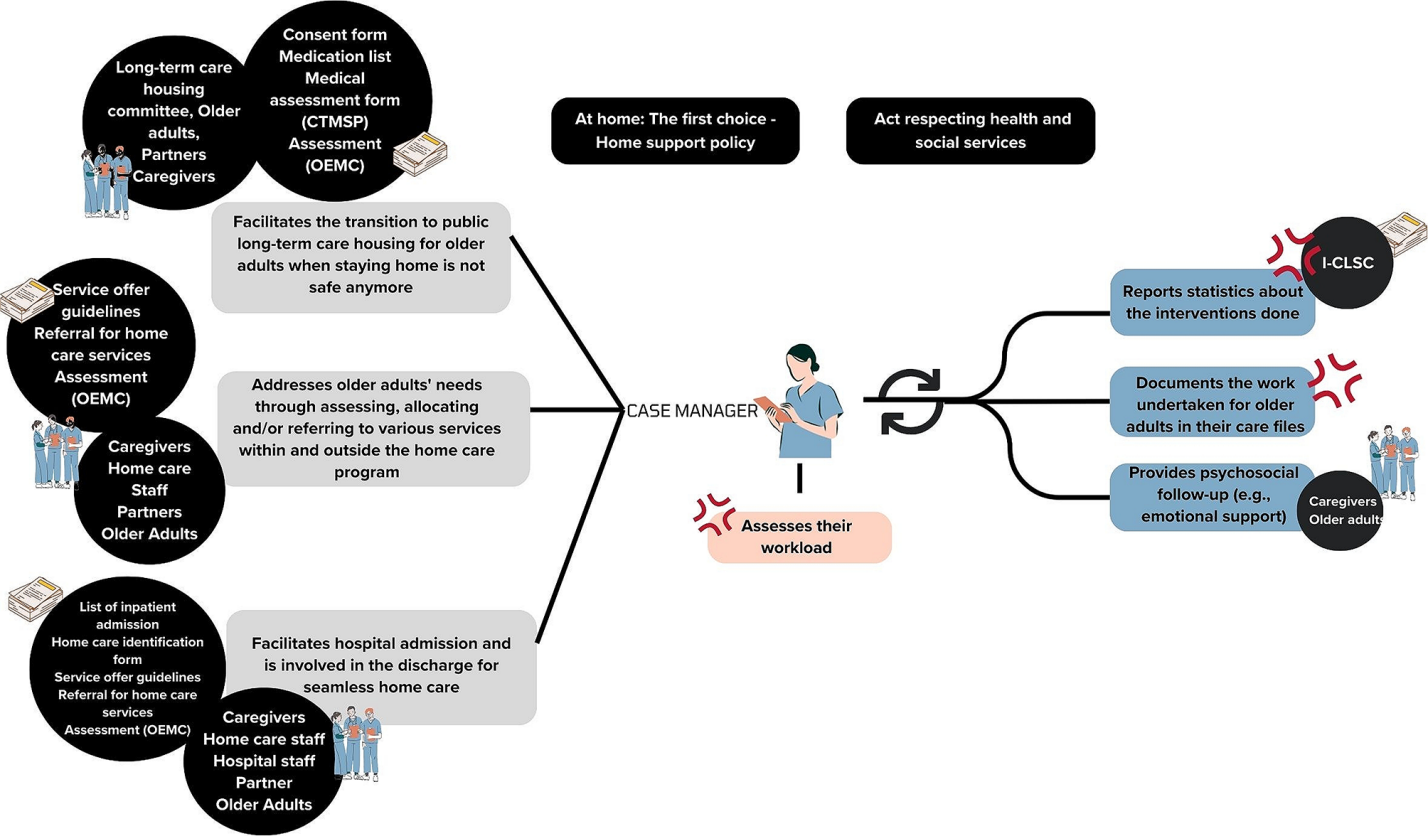
The main tasks of being a case manager. Legend. The black rectangles represent the boss texts identified during the first level analysis. They are placed above all the main tasks, as we suspect they regulate the work of case managers. The grey rectangles are the main clinical tasks, which include direct and indirect tasks. The blue rectangles represent the tasks that must be done continuously depending on the action done by case managers. These can be clinical or non-clinical task. As they are recurrent, they are not shown in Figs. 2, 3 and 4, unless relevant. The pink rectangle represents a periodical non-clinical administrative task. The circles represent the people case managers interact with while doing these tasks and the documents they use to do their work. The red symbol represents where tensions were found

**Fig. 2:**
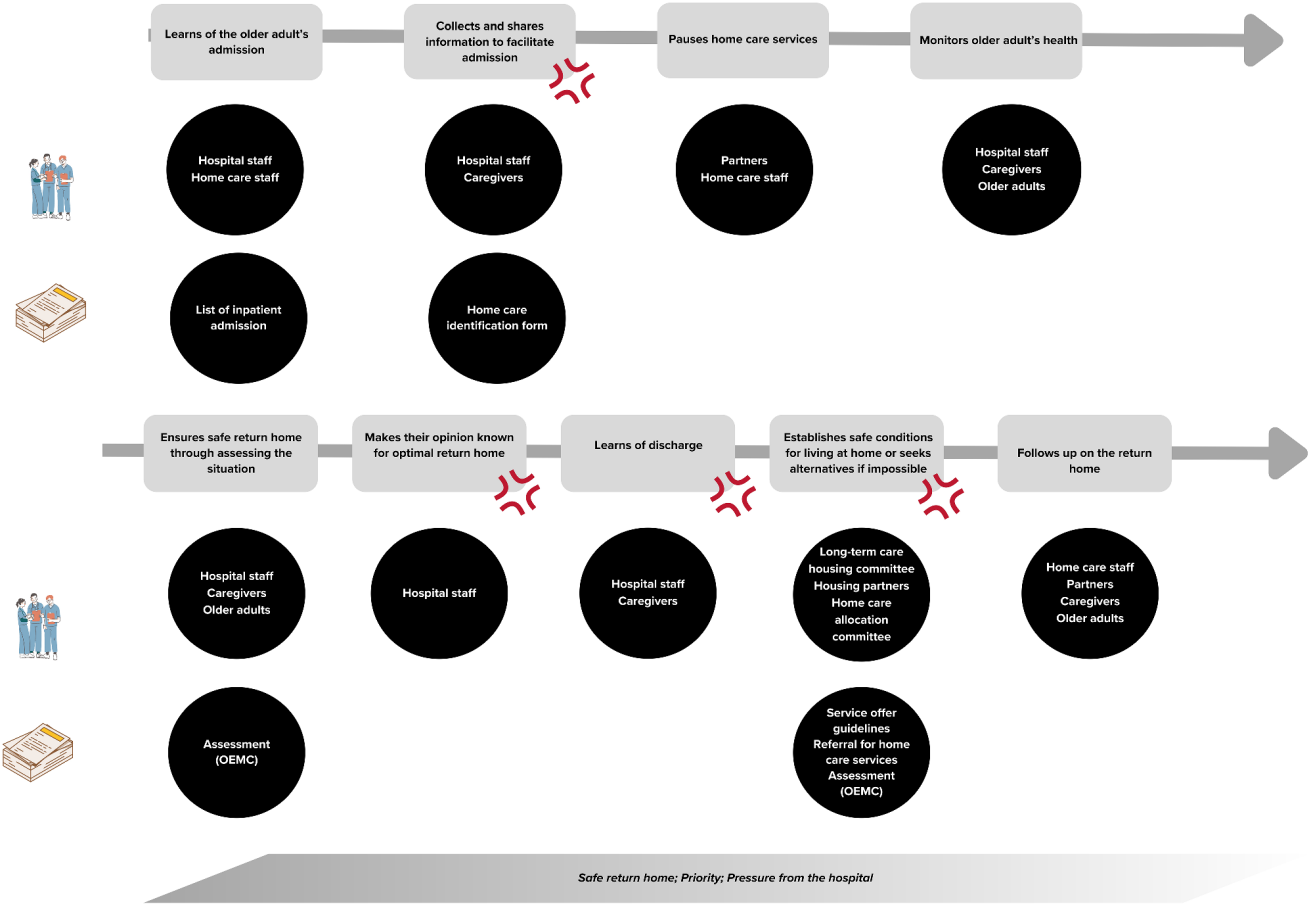
Facilitating inpatient admission to hospitals and being involved in managing the discharge process to resume home care. *Note* The legend here applies to Figs. 2, 3 and 4, inclusively. *Legend*: The grey rectangles are the main clinical tasks, which include direct and indirect tasks. The circles represent the people case managers interact with while doing these tasks and the documents they use to do their work. The red symbol represents where tensions were found. Words written in the parallelogram are recurrent words used by case managers

**Fig. 3:**
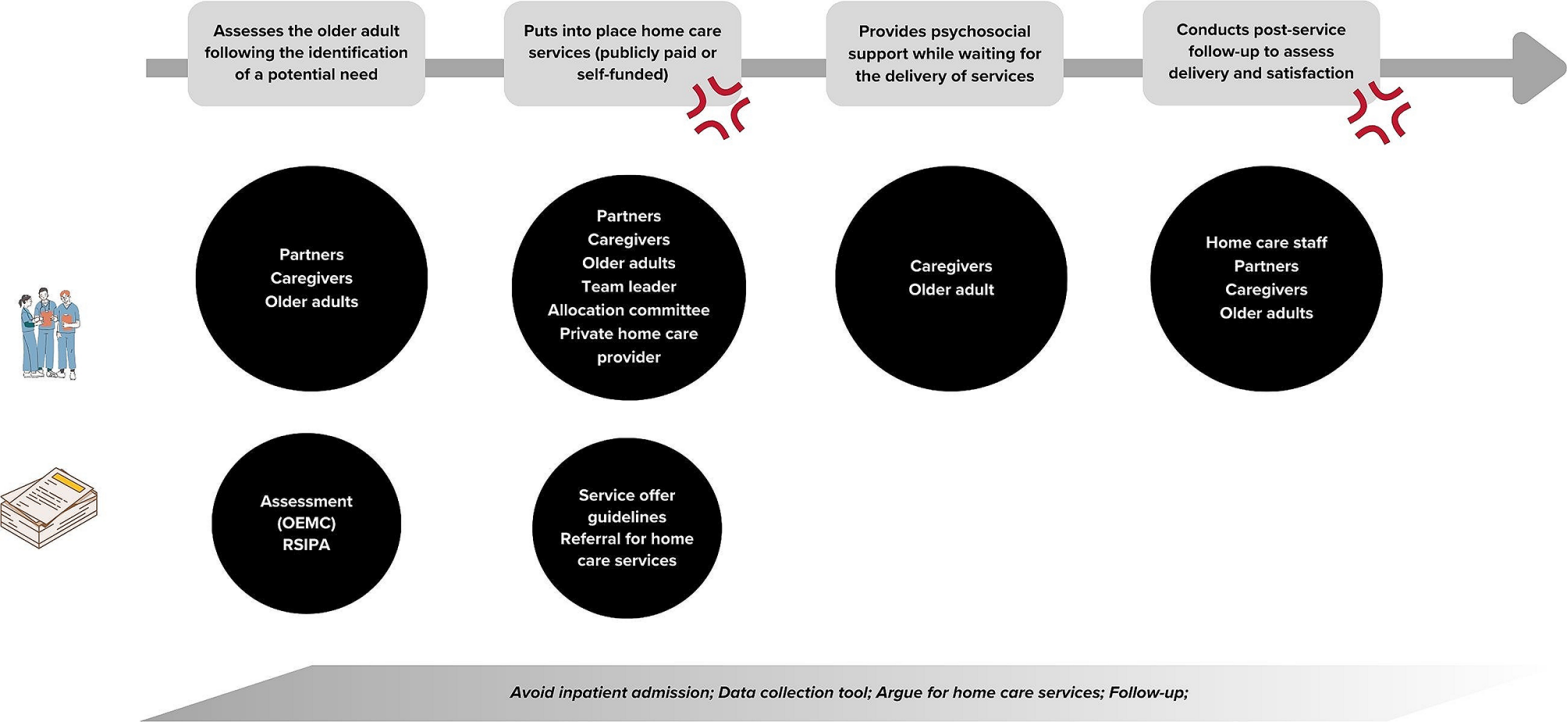
Assessing the needs of older adults and intervening accordingly

**Fig. 4:**
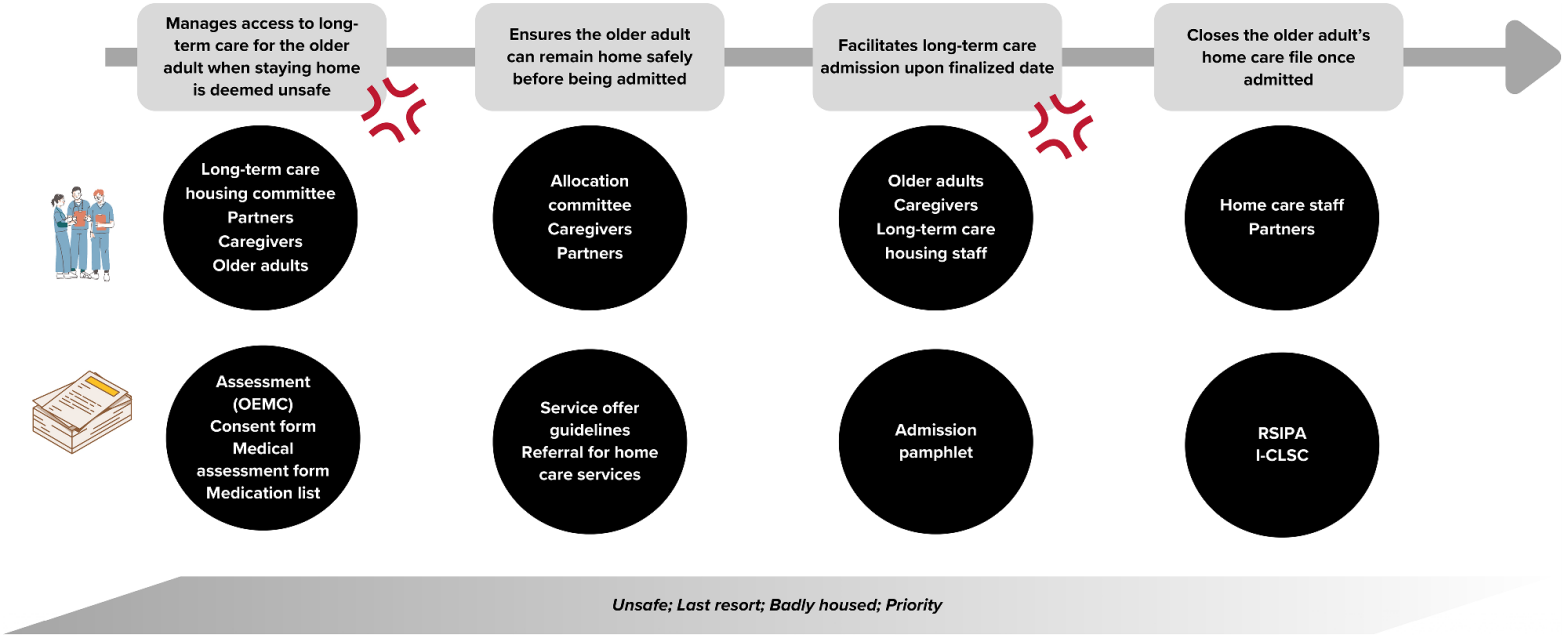
Supporting the older adults’ transition to public long-term care housing when staying at home is deemed unsafe

This section presents the work and the tensions inherent to the work of case managers, from their standpoint. Figure 1 represents the main tasks of a case manager. Figures 2, 3 and 4 present the main sequences of work. Table 3 presents all the tensions identified through data collection. The tensions remaining after the survey are presented and explained in detail in Table 4. All three tensions reported in Table 4 were shared by case managers, despite their various training, years of experience and gender.

**Table 3:** Tensions presented in the survey

**Returning older adults at home safely vs freeing up hospital beds** I should be insisting on lengths of stay that allow a safe return home for the older adults in my case load. However, I feel I am helping to empty the hospital beds as quickly as possible.
**Respect the “complex” case management model and the “regular” case management model vs. promote stability for users** ^1^ **For “regular” case managers:** I should transfer my files that have become “complex” to the case managers dedicated to these files. However, I’m keeping my files because I think it’s better for older adults to have “stability”. **For “complex” case managers:** I should receive the “complex” files from “regular” case managers. However, although I believe that these files would require my intervention, I don’t insist on receiving them because I feel that this offers stability to older adults.
**Using home care allocation documents in the prescribed manner vs. entering information in the documents to arrive at the allocation I deem necessary** I should complete the documents in the prescribed manner. However, to obtain the necessary services for older adults, I “manipulate” the information in the documents so that it matches the criteria leading to the allocation of needed home care services (e.g., the older adults in my caseload are all “poor”).
**Respecting organisational rules and not being in a position to enable ageing in place vs. transgressing them to enable ageing in place** I should follow certain rules in my work. However, I sometimes break them (e.g., I go shopping with the older adult, I give them a ride in my car, I do things I shouldn’t do) to enable them to age in place.
**Delegating care to “partners” vs managing the quality of services provided by partners** I should be working with my partners. However, I feel that my work with them is mainly aimed at controlling the quality of the services they provide and “putting out the fires” they cause.
**Meeting organisational requirements vs spending more time providing care for older adults** I must meet organisational requirements (e.g., up-to-date notes, up-to-date statistics, number of up-to-date assessments, workload assessment). However, in meeting these organisational requirements, I reduce my involvement with older adults.
**Following the normal procedure for allowing transitions to long-term care homes vs transgressing the procedure for a patient-centered outcome** I should stick to the transitions to long-term care homes formal processes. However, by respecting this process, I feel that older adults would end up in long-term care homes that do not correspond to their needs (e.g., far from their informal caregivers) and would experience a dehumanizing process. As a result, I transgress the process (e.g., filing a request when it’s not yet time for transitioning, changing older adults from one home to another so they can get their first choice, which requires me to break confidentiality rules).

1. This tension uses “regular” case management and “complex” case management. In this I[U]HSS, the case management model had been changed previously to data collection (from regular to complex). In the complex model, some professionals and technicians were supposed to have older adults in their caseload that would require less service coordination and some professionals would have older adults in their caseload that would require “intense” coordination of services. However, in practice, their work was the same

**Table 4:** Tensions reported by over 50% of standpoint informants as regularly or always encountered during work

Tensions	Description
Returning older adults at home safely vs. freeing up hospital beds	Case managers strive to ensure the safety of older adults in their homes. However, they perceive pressure from the hospital staff to swiftly arrange home care services for a prompt discharge of older adults.
Delegating care to “partners” vs. managing the quality of services provided by partners	Case managers should only be minimally involved when care is provided by a partner. However, in practice, they end up overseeing the quality of the services as well as dealing with scheduling issues.
Meeting organisational requirements vs. spending more time providing care for older adults	Case managers must meet organisational requirements (e.g., up-to-date older adults’ file, up-to-date reporting of the clinical work they do through statistics, up-to-date mandatory assessment). However, in meeting these organisational requirements, they are reducing the time they are directly involved with older adults, thus the quality of care they can provide.

### Returning older adults at home safely vs. freeing up hospital beds

Case managers feel pressured to respond to the needs of the hospital, as opposed to focusing on the needs of older adults in home care. Indeed, case managers’ daily tasks are significantly impacted by the hospital admission or discharge of any older adult in their care. Thus, they perceive that their work mostly aims to help physicians in the hospital to free up beds, rather than putting into place the necessary conditions for a safe return home following discharge:

*“It’s an upside-down world. […] When I say upside down, I mean when you receive the list of older adults hospitalised, you have to follow up [if one older adult in your caseload is hospitalised]. Well, if you do not, they’re going to call you. […] That means it becomes a priority. […] We’re not doing prevention [work], we’re doing posttreatment. Because they’re going to be worried about when we will take the older adult out [of the hospital]. Basically, because they can’t stay in the beds for too long.”* (W173561, interview).

Given the significance of hospital-related events, all case managers initiate their day by following a consistent routine in the home care office. They access the list of hospitalised older adults provided by their local hospital. Their objective is to identify whether any of the older adults under their care have been admitted to the hospital. Additionally, they check their voicemails in case the hospital staff have reached out for further information or if there are indications of an impending discharge. The occurrence of an inpatient admission or the possibility of a discharge often necessitates case managers to deviate from their preplanned schedules. For instance, home care visits may need to be rescheduled. This could be a contributing factor as to why many do not plan their days in advance: *“[…] in case management, it is very different from one day to the next. I do not plan so much in advance because things move around a lot*.” (W385214, interview).

When the case managers learn that one of the older adults in their caseload has been admitted to the hospital, their main tasks involve notifying the hospital staff that they are their designated case manager by sending a form and calling them, as well as informing other home care workers involved in the older adult’s care. Additionally, they temporarily suspend home care services during the hospital stay. Throughout this period, case managers may receive multiple phone calls from hospital staff inquiring about the readiness of home care services or the health of the older adult, prior to hospitalisation. It appears this situation arises because hospital staff seem to prefer contacting case managers rather than looking at the older adults’ file in the software utilised by home care services. Despite the frequency of these calls, some case managers have expressed that their recommendations for a safe return home are often disregarded, unless they actively participate in the interdisciplinary meetings held directly at the hospital:

*“I had already phoned when he was under observation to say he was not well. […] However, as long as you do not show up at the meeting and speak directly to the team, it is like nobody’s heard anything, it is truly peculiar. […] We truly have to get in there and name things in front of the whole team. Otherwise, it appears that there’s nothing, the information never gets out.”* (W336925, interviews).

Furthermore, due to communication challenges between hospitals and home care services, case managers often learn the discharge of older adults at the last minute. In those situations, if there is a need to add or modify home care services, case managers must expedite the approval process with the relevant personnel. This may involve (1) requesting home care services through the “emergency process,” (2) engaging in discussions with their team leaders in home care to gather support for expediting the home care allocation process or to ask them to put pressure on the hospital for delaying the discharge, or (3) contacting the Allocating committee, that is, the committee deciding of the allocation of home care, to prioritise the request of home care services for older adults. Additionally, case managers can be pressured to put home care services in place quickly by a specific employee in the home care programme whose role is to follow up on the transition from the hospital to the home. As they become accustomed to being pressured to ensure the return home from the hospital is done quickly, some case managers anticipate the hospital staff’s response and take preventive actions, based on their expectations. For example, some visit the hospital to obtain the necessary forms for the next tasks (e.g., long-term care housing), even if they were not officially informed of the discharge.

### Delegating care to “partners” vs. managing the quality of services provided by partners

Home care programmes establish partnerships with service providers to deliver home care services. Ideally, case managers should only be minimally involved when care is provided by a partner. They should intervene when challenges arise (e.g., falls or difficulties in providing care as determined in the care plan). In those situations, case managers are expected to work with these partners to identify and implement solutions. However, in practice, rather than delegating care, case managers often focus primarily on ensuring the quality of services by becoming involved in managing the services, as well as in scheduling issues. This dynamic is sometimes present for private senior housing providers and is particularly evident when dealing with agencies. Agencies are private organisations paid by public organisations (such as I[U]HSSS) to provide workers to deliver allocated home care services.

During observations, various issues were identified with services provided by partners. These included partners showing up for home visits that were not scheduled and were already being provided by another partner, or simply not showing up for scheduled visits. Furthermore, some partners neglected the care of older adults by leaving them in incontinence briefs for extended periods of time. Others failed to inform case managers when older adults fell. When partners deviate from the agreed-upon arrangements, case managers often find themselves burdened with additional work. For instance, if partners decide that they no longer want to work the allocated hours, case managers need to submit a new request for home care services to the Allocating Committee, resulting in increased paperwork. Furthermore, when faced with difficulties in providing care to older adults, partners tend to be reluctant to implement suggested solutions proposed by case managers. In some instances, agencies may choose to discontinue their services when confronted with complex situations: “*Agencies, when they do not like it, they leave. They can go one day and say, “I’m not going there anymore,” then they leave. Whereas the community organisations, they work with us. The girls who work for the community organisations, they want to do well, they will persevere a lot more.*” (W385214, interview).

Case managers may opt for alternative methods of providing home care instead of relying on partner agencies. For instance, some make it a priority to explore options such as utilising a consumer-directed approach. This allows the older adult to hire someone they know or someone from a list of employees provided by the home care organisation, without paying for out-of-pocket care: “*I hate dealing with agencies. I’m very good at finding employees to work with families. So, I use the chèque emploi service programme [name of the consumer-directed approach] a lot*” (W398765, interview).

Based on their past experiences with partners, case managers have also developed various strategies to enhance the quality of care provided. One such strategy involves creating highly detailed care plans. For example, they may specify the exact steps to be taken, such as applying toothpaste to the toothbrush when dental care is needed. Another strategy employed is to personally meet the employees from partnering organisations for their first home care visits: “*I like to go and introduce myself at the time of a visit, to meet the employee who’s there. What I always explain to families and older adults is that it is the home care organisation that sends this person, and I’m somewhat responsible for the person who’s sent there*.” (W385214, interview). By being present during these visits, case managers can actively monitor and assess the quality of care being delivered. When faced with different situations they cannot do anything about, they resort to advising older adults to lodge a formal complaint and report the situation to their team leaders.

Finally, as the partners are not part of their organisations, case managers often face challenges in effectively giving and obtaining information from them. For example, communications must go through their administrative agent, which will then contact the agencies. With community organisations, they have access to the administrative staff but rarely to the workers who provide the care. As such, case managers may encounter difficulties in receiving timely updates about older adults’ well-being and any incidents that may occur, such as falls or visits to the emergency room. To gather information about these situations, case managers rely on their home care visits. They will actively engage with older adults and their informal caregivers, asking questions and seeking insights to better understand the care provided by the partners and any notable incidents that may have occurred. As communication is also difficult with private seniors’ residences, case managers also use home care visits to directly speak with the staff to gather information about older adults.

### Meeting organisational requirements vs. spending more time providing care for older adults

Case managers are bound by organisational requirements. One of these requirements is that they must meet a target number of interventions each day for older adults. These interventions, called “statistics”, are recorded, and tracked through software. The case managers themselves input various codes that denote the clinical actions undertaken. These codes are defined in the I-CLSC guidelines (*Cadre normatif I-CLSC*), which are province-wide guidelines used to support home care staff, managers, and medical archivists when working with the statistics. Each reported intervention involves one to three codes that reflect both direct and indirect clinical actions, along with the duration of these actions. Table 5 gives examples of the recurrent statistics reported by case managers observed during data collection.

**Table 5:** Example of statistics

	Example of the observed action	Associated I-CLSC code
**Direct clinical actions**: In the presence of the older adult and/or the informal caregivers		
	Assessing the older adult	6500 Assessment or re-assessment
	Observing and collecting information about a specific situation	6700 Observation or follow-up actions (physical, psychological, psychosocial)
	Psychological follow up	7700 Psychosocial actions
**Indirect clinical actions**: Without the older adult and/or the informal caregiver		
	Engaging in administrative procedures for the older adult	6000 Paperwork for accessing home care services outside of the home care programme
	Meeting other professionals involved with an older adult to solve a problem	9100 Clinical consultation and related care coordination

The statistics are only used for management purposes, and as such, have no clinical value. Case managers must meet a specific number of interventions per day that are reported through the statistics because these statistics are utilised to calculate performance indicators. In return, these performance indicators are linked the funding provided to home care programmes by the Quebec government. However, case managers perceive these statistics as inadequate, since they cannot report the main tasks they engage in, particularly the exchange of information or all the tasks that require writing about the older adult’s situation.

In addition to statistical reporting, case managers must document the work they undertake for older adults in their care files. For example, they need to document when they talked to the older adult, what was said, what are the next steps. If they talk to someone involved in the older adult’s care, they also need to document who they talked to, what was said, what are the next steps, etc. This needs to be documented on specific time frames, as determined not only by their organisation but also in accordance with professional standards.

Furthermore, case managers must assess their workloads. To do so, they need to evaluate the workload associated with each individual older adult in their caseload through an Excel grid. The whole process and the documents that are used are based on one specific document produced by the social workers’ licensing body. This assessment involves assigning a score to each older adult of one’s caseload, based on specific criteria highlighted in a document named Complexity ratio. The criteria used to assess a score on the Complexity ratio document may include the number of actions needed, available resources (financial, material, social), frequency of contact with the older adults, risks associated with living at home, and more. The scores of all older adults in their caseloads are then added up to determine an overall value. Each case manager must attain a predetermined caseload overall value. If the resulting value is below the target, case managers may need to take on additional older adults. However, most case managers are unable to attain the targeted value. To them, this is due to the fact that they must fill out too much paperwork. Those who do attaint the target value often tend to be overloaded with paperwork and have little time to spend with older adults.

Finally, case managers must complete an extensive mandatory assessment of the older adult once a year or when the condition of the older adult changes drastically and as such, could impact eligibility for home care services. All this is done using a standardised assessment tool (OEMC). The assessment has three main limitations for case managers. First, is it time-consuming, due to the extensive number of questions and all the writing required. As they feel they rarely use the information collected in their work, they do not see this tool as a genuine assessment. Second, case managers also feel the tool focuses on ensuring that older adults fit into predefined criteria rather than considering their situation holistically and systemically. Thus, the tool is limited in its ability to identify the real needs of older adults (i.e., the tool will suggest in-home respite care without considering the willingness of the caregiver to receive this service). Third, with the other administrative tasks, the time it takes to fill out the assessment does not leave them the opportunity to conduct an assessment from their disciplinary training, which also does not seem to be a priority for their managers. However, case managers find this confusing as they are hired to be regulated professionals and as such, should engage in profession-specific tasks. Additionally, the organisational importance given to the completion of this assessment has two consequences for case managers. First, some case managers feel like their visits to older adults are only motivated by the necessity to complete the tool. As such, they do not understand that they can perform a home care visit just to follow up on the psychosocial situation of the older adult between the yearly re-assessment. Second, other case managers feel the yearly assessment is irrelevant, as it is only useful as a performance measure.

All the paperwork related to meeting organisational expectations is time-consuming: *“We do paperwork approximately 70% [of our time,] and 30% [of our time] is spent with older adults.”* (W155114, obser-view). Thus, case managers feel that the time they can spend with older adults providing quality care is limited. As time is limited, case managers find it difficult to develop relationships with older adults, which are essential to provide services that respond to their needs. Relationships are also strengthened when services are provided. However, resources are becoming scarce in home care, due to staff shortages. As such, case managers deal with dissatisfaction from older adults as well as their caregivers, as they are the point of contact for home care: “*I have little time, since November 2022, to create a relationship with older adults. All I do is manage crises; therefore, the bond created with older adults, or their family, relies on obtaining the services they want. I have no control over this. So, my relationship with older adults depends on obtaining services. […] so, when they don’t get what they want, I am the one dealing with their dissatisfaction, as I’m the entry door to home care*” (survey comment). Experienced case managers recognise that over time, increasing organisational expectations makes it difficult to perform home visits.

Despite needing to meet organisational requirements, many case managers prioritise providing care and supporting older adults rather than dedicating extensive time and effort to reporting their day-to-day work. Through observations, it has been revealed that they only report the minimal number of statistics asked per day. Additionally, as many actions can count as statistics, to avoid wasting time finding the right name for the action they did, some will only report the actions that have names that they are familiar with, thus underreporting other actions. Others may choose to postpone documenting the older adults’ situations in their files until it becomes necessary, such as when it is time to arrange long-term housing. Regarding the assessment tool, many case managers only utilise the tool when necessary (e.g., requesting long-term care housing or specific services) rather than collecting, analysing and writing the assessment only to meet organisational expectations.

## Discussion

We identified three tensions within the role of case manager. They are (1) returning older adults at home safely vs. freeing up hospital beds; (2) delegating care to “partners” vs. managing the quality of services provided by partners; and (3) meeting organisational requirements vs. spending more time providing care for older adults. Our findings are in line with those of Benoit et al. [68], who surveyed 697 home care workers (i.e., professionals and technicians) in Quebec. In this study, they explored how home care workers view the purpose of home care services and their perception of how their managers and the government viewed this purpose. According to these home care workers’ perspectives, their managers and the government place greater emphasis on meeting performance targets and reducing hospital bed occupancy. Conversely, the home care workers believe that their work should focus on delivering high-quality home care services tailored to the specific needs of older adults. These elements are all part of the tensions we identified in our study. Indeed, case managers need to work closely with the hospital and its partners. They are accountable to the Ministry of Health and Social Services. They encounter ethical dilemmas regarding the quality of care provided by partners. They must meet organisational expectations through performance targets. These aspects evoke the three lenses of professional practice context analysis, that is, accountability, ethics, and professional-as-worker, which will be discussed below. Finally, our discussion will then conclude on power in home care.

Our findings show how the three lenses of professional practice context analyses, namely, accountability, ethics, and professional-as-worker [69], can be used to shed light on the tensions case managers experience in their work. Accountability refers to whom case managers answer to and for what obligations [69]. Indeed, as professionals, through their licensing bodies, case managers are accountable to the State to ensure the quality of the services, but also to their employer, service funders, clients and families, and colleagues [70]. Ethics refers to the balance between two values (e.g., quality of services vs. access to services) that case managers are confronted with [69]. This lens is of importance as professionals must put their knowledge to the service of the older adults, which in this situation, are vulnerable as they do not have that knowledge [71]. Professional-as-workers refers to the actual organisational conditions (workload relative to resources available) that enable (or not) professionals to accomplish their mandate while maintaining their well-being [69]. This is because work organisation (e.g., workload, control, support), might influence well-being at work [72].

Regarding accountability, one tension is related (1) to the number of interventions case managers must reach each day by reporting them as statistics and (2) the completion of yearly assessments. Both these are performance measures used by the Ministry of Health and Social Services. Indeed, case managers have to report their actions with older adults using a software system, but they feel this takes away valuable time that could be spent providing care, which is in line with other studies [73, 74]. Additionally, case managers report that the yearly assessment takes time to complete and does not allow a genuine assessment. As professionals, case managers are accountable to their licensing bodies. Furthermore, they are hired by the organisation as professionals and are therefore also accountable to this organisation through that role. However, due to the overwhelming number of administrative tasks, including handling statistics and conducting yearly assessments, case managers are unable to perform formal assessments aligned with their disciplinary training [75]. This puts them at risk of disciplinary sanctions. Additionally, they become confused, as they are unsure of what role they should take (professional and conduct their own assessment or case manager and conduct the mandatory one). Despite being an organisational requirement, meeting the performance measures through the completion of the assessment does not guarantee good care for older adults. Indeed, documented issues related to accountability, both in terms of statistics and mandatory assessments, include ethical dilemmas [76], suffering [77], reduction in the quality of provided services [57], reduced ability to respond to older adults needs who lack complementary coping resources [39], fewer services provided [38], and difficulties meeting the needs of older adults outside the assessment tools [78–80]. Thus, this could explain why case managers reported only the minimum number of statistics and only took time to write down the assessment when necessary. However, no studies directly document the connection between statistics about the work, completion of an assessment tool by case managers, performance measures, and professional obligations. As such, the links among these aspects remain unclear.

In terms of ethics, case managers often experience a tension in which they feel compelled to handle the lower quality of services provided by their partners instead of delegating care to them. The relationship with partners raises ethical concerns, as collaboration becomes challenging, due to the unequal nature of the partnership. Consequently, when situations require that partners be involved, case managers find themselves torn between two choices: (1) going beyond their role to ensure the delivery of quality services, thereby contributing to an unequal relationship, or (2) refraining from intervening, resulting in suboptimal care for older adults. However, the latter choice raises concerns regarding quality and access to care. Older adults receiving home care in Quebec have reported opting for no services at all rather than receiving poor-quality services from partners [16]. To navigate this ethical dilemma, case managers employ various strategies, such as managing the quality of care. Some strategies involve direct influence on the partners’ work, such as developing detailed care plans and meeting with the partners. Other strategies do not always directly affect their partners, such as advising older adults to file complaints and communicating with their team leaders. Additional studies conducted outside of Quebec also show a trend towards contracting in home care [81–86], that is, when a health and social service organisation, rather than providing the service itself, entrusts a partner to provide a specific service in exchange for payment [87]. In many cases, contracting can pose challenges to the delivery of quality services [83–85]. As seen in our study, these situations generate additional administrative tasks that should not be part of the case managers core responsibilities, diverting attention from the essential tasks of case management. Ultimately, this could impede case managers from focusing on keeping older adults home safely. Thus, as regulations play a major role to improve the quality of home care service in for-profit care or contracting [88], our research highlights the necessity for interventions aimed at enhancing the quality of care, which would, in turn, ensure genuine access to services.

With the professional as a worker’s lens, the expected caseload assigned to case managers appears to be unrealistic. In fact, many of them fail to reach such caseloads, and those who do end up with limited time to dedicate to older adults. The lack of time for older adults is supported in a recent report from the organisation in charge of assessing the performance of Quebec’s Health and Social Services organisations, as well as by the scientific literature [5, 24]. To address this issue, case managers have resorted to minimising paperwork as much as possible. They seem to have implemented this workaround to ensure meaningful service delivery while meeting organisational expectations and safeguarding their own well-being at work [89], since workload [90, 91] and time pressure [92] are related to burnout for home care professionals. However, studies conducted in other countries with case managers show that they tend to spend a lot of time on administrative tasks, including documenting the older adults’ situation [24, 93], which is important for quality care [94–96]. Thus, this workaround raises challenges potentially impacting both service quality for older adults and the well-being of case managers.

Consequently, using the three lenses of professional practice analysis allowed to highlight two key points of our results, as this model can be used to interpret tension in professional practices [69]. First, case managers’ well-being could be diminished, and second, so could the quality of the services. Thus, our study serves as a first step for identifying where to intervene for better well-being for case managers, as well as better quality services in home care [69].

Finally, it also appears that case managers experience limited power in their daily role, despite occupying a privileged position in home care [53]. As they have to complete the necessary assessment according to specific timeframes, they become subordinate of their team leaders, managers, and the Ministry of Health and Social Services [80]. Additionally, while they do possess the ability to refer clients to other services, thereby carrying significant influence over the work of other workers in home care [53], this influence appears to be confined to the boundaries of the home care programmes. Indeed, case managers’ work is greatly influenced by the hospital and the “partners,” that is, organisations situated outside of home care programmes. For instance, case managers must be readily available to address inquiries and to ensure home care services are ready following early discharges requested by hospital staff. In dealing with the partners, case managers perceive that they bear the responsibility of potential substandard care and need to intervene when inadequate situations arise. These results are in line with those of many studies documenting work in home care. The power relations between case managers (or other health professionals) and the hospital are documented abundantly but not always directly (e.g., [12, 33, 97–100]). For example, case managers tend to focus on discharge issues [33, 97]. Power issues with partners are also discussed indirectly in the literature on home care (e.g., [82, 83, 85, 101, 102]). These issues include partners not providing the planned care [82, 102], difficulties in collaborating [83] and lesser quality services [82, 101]. However, the available literature does not focus on power issues between partners and case managers. Thus, considering that power relations impact the care provided to older adults [53], our study contributes to important and new insights related to power relationships in home care.

### Recommendations for practice and research

Considering our results, two tensions require recommendations. First, it is essential to align the choice of statistics as performance measure with day-to-day work. These statistics should also be in line with the specific needs of older adults, as a gap exists between the needs identified by professionals, and those identified by older adults [62, 103]. Furthermore, compiling statistics should be done without taking time away from older adults. Thus, incorporating the older adult’s file information as a statistic would serve the dual purpose of reducing the time spent on reporting statistics while ensuring the inclusion of necessary information for optimal care coordination. Additionally, as the links between statistics, mandatory assessments and performance measures remain largely unexplored, future research is needed.

Second, regarding work with partners, clear guidelines need to be established, outlining the scope of case managers’ responsibilities as well as what constitutes good care. This could help clarify case managers’ roles in ensuring quality care provision and what constitutes high-quality care.

### Strengths and limitations

This study has several strengths, primarily attributed to the constant validation of the analysis with standpoint informants. This validation involved multiple methods, including the use of obser-views following observations, interviews to confirm the mapping of the sequences of work, and a survey for the identified tensions. Moreover, data triangulation was achieved through the inclusion of different professions and diverse data collection methods. Additionally, first, second and third authors lack professional experience in home care, which could be seen as a limitation. However, as institutional ethnography wants to uncover taken-for-granted work and pays attention to the language [104], having a novice perspective towards these elements allowed us to ask for clarifications in a natural, nonexpert language, rendering the taken-for granted work visible [35, 105]. At the same time, the last author has 10 years of experience in home care practice in Quebec and checked the analysis. This combination of different levels of knowledge allowed the team to ensure that no relevant aspects were missed.

It is also important to acknowledge two limitations. First, this study is grounded in a single perspective, that of case managers. Thus, we lack the perspective of hospital staff, partners, managers, and team leaders, which are all actors influencing the case managers’ work. Second, it is possible that case managers engaged in post hoc rationalisation when questioned about their everyday work. To mitigate this risk, we sought practical examples from them, based on their day-to-day actions, whenever possible.

## Conclusion

Case managers’ work allows older adults with multiple healthcare needs to age in place. However, case managers seem to encounter tensions in their work, that is, disjuncture between what they are supposed to do and what they actually do. Thus, we aimed to describe the tensions encountered by case managers in home care programmes for older adults in Quebec and their influence on day-to-day work. We found three main tensions in their work. First, case managers sense that their work is focused on helping free up hospital beds instead of making sure older adults return home safely. Second, case managers feel the need to compensate when poor quality care is provided by partners instead of being able to delegate this work. Third, they spend less than the desired amount of time with older adults because they must meet organisational expectations through administrative tasks. By employing the lenses of accountability, ethics, and professional-as-worker, we have identified significant challenges faced by case managers and derived actionable recommendations for practice, which could act as a lever for quality care and research. First, as case management requires considerable time away from older adults, organisational requirements should be revised to increase the time actually spent with them. Second, clear guidelines regarding how to work with partners should be established to deal with the ethical dilemmas encountered. Third, future research should document the link between statistics, the use of mandatory assessments, and performance measures, as this remains unexplored. Finally, our research suggests that, despite their privileged role in home care, case managers seem to have limited power with the hospital and partners.

### Electronic supplementary material

Below is the link to the electronic supplementary material.

**Supplementary Material 1:** The page 1-2 of supplementary materials presents the sociodemographic questionnaire. The page 3 presents the observation grid. The pages 4-7 present the interviews guide

## Data Availability

All data generated or analysed during this study are included in this published article.

## Abbreviations

I[U]HSSC: Integrated [University] Health and Social Service Centres
SAPA: Support Programme for the Autonomy of Seniors (*soutien à l’autonomie des personnes âgées)*
