# Supplementary material for: Tensions experienced by case managers working in home care for older adults in Quebec: first level analysis of an institutional ethnography
Source: BMC Health Serv Res. 2024 Mar 6;24:296. doi: 10.1186/s12913-024-10709-6 (PMC10918893; doi:10.1186/s12913-024-10709-6)
Supplement: Supplementary file 1 — Supplementary Material 1: The page 1-2 of supplementary materials presents the sociodemographic questionnaire. The page 3 presents the observation grid. The pages 4-7 present the interviews guide [file 12913_2024_10709_MOESM1_ESM.docx]

# Sociodemographic questionnaire

Case manager ID: __________

1. Gender:

| 🞎 | Man | 🞎 | Woman | 🞎 | Other | 🞎 | Prefer not to say |
| --- | --- | --- | --- | --- | --- | --- | --- |

1. Year of birth: __________
2. Are you member of a professional association?

| 🞎 | Yes | 🞎 | No |
| --- | --- | --- | --- |

- 1. If so, since when? ____________
  2. If so, which professional association are you a member of?

| 🞎 | Québec Order of Social Workers and Marriage and Family Therapists |
| --- | --- |
| 🞎 | Québec Order of Nurses |
| 🞎 | Québec Order of Occupational Therapists |
| 🞎 | Québec Order of physiotherapy |
| 🞎 | Other (specify): ___________________________________ |

1. What is your current position? ______________________________________________
2. What is your total number of years of work experience **in your current position**?

| 🞎 | < 1 |
| --- | --- |
| 🞎 | 1-4 |
| 🞎 | 5-9 |
| 🞎 | **≥** 10 |

1. What is your total number of years of work experience in **home care**?

| 🞎 | < 1 |
| --- | --- |
| 🞎 | 1-4 |
| 🞎 | 5-9 |
| 🞎 | **≥** 10 |

1. Do you have management experience in the health and social services sector?

| 🞎 | Yes |
| --- | --- |
| 🞎 | No |

1. Identify the various practice settings in which you have worked as a professional member of your order (check all that apply).

| 🞎 | Hospital, internal | 🞎 | Industry / Retail |
| --- | --- | --- | --- |
| 🞎 | Ambulatory centre/outpatient clinic/day hospital | 🞎 | Residential and long-term care centre |
| 🞎 | Local community health centre | 🞎 | Physical disability rehabilitation centre |
| 🞎 | Day centre | 🞎 | Alcohol and drug rehabilitation centre |
| 🞎 | Rehabilitation centre, intellectual disability | 🞎 | Placement agency |
| 🞎 | Youth centre | 🞎 | School / school board |
| 🞎 | Private clinic | 🞎 | Governmental / paragovernmental organisation |
| 🞎 | Childcare centre | 🞎 | Research centre |
| 🞎 | Independent practice | 🞎 | Community / association groups |
| 🞎 | Post-secondary educational institution | 🞎 | Other :______________________________ |

1. What is the highest level of education you have completed?

| 🞎 | College | Specify program name: __________________________________ |
| --- | --- | --- |
| 🞎 | Baccalaureate | Specify program name: __________________________________ |
| 🞎 | Master | Specify program name: __________________________________ |
| 🞎 | PhD | Specify program name: __________________________________ |

1. Indicate, if applicable, any other training you have taken (other than that already indicated).

| 🞎 | Trades | _____________________ | 🞎 completed | 🞎 in progress | 🞎 not completed |
| --- | --- | --- | --- | --- | --- |
| 🞎 | College | _____________________ | 🞎 completed | 🞎 in progress | 🞎 not completed |
| 🞎 | Baccalaureate | _____________________ | 🞎 completed | 🞎 in progress | 🞎 not completed |
| 🞎 | Master | _____________________ | 🞎 completed | 🞎 in progress | 🞎 not completed |
| 🞎 | PhD | _____________________ | 🞎 completed | 🞎 in progress | 🞎 not completed |
| 🞎 | Other | _____________________ | 🞎 completed | 🞎 in progress | 🞎 not completed |

# Observation grid

Case manager ID: __________

Date: __________

| Context: | People: | Activities: | Questions: |
| --- | --- | --- | --- |
| Where does the observation take place? When? | Who is there?  Why?  How are these people connected? | What are the people doing? | What needs to be explored further?  Are there any particular words used? |
|  |  |  |  |

# Interview grid – home care program with observations

Based on observations and informal discussions, I have identified several areas I'd like to explore with you today. Please note that there are no right or wrong answers to my questions. I simply want you to tell me what you know about these areas. This interview will be divided into three parts.

***Part A****: questions about the language you used*

***Part B****: questions related to the main tasks you do*

***Part C****: meeting conclusion / other points not covered, as requested by you*.

Follow-up questions:

- Explain to me with examples of actions (or tasks) you need to perform?
- (Who) or (What document/regulation/policy/procedure), if any, would be likely to give me more information on [insert area discussed]?

***Part A : language used***

*Note. in quotation marks (“”) are the words used by case managers*

Ageing in place and safety

How do you define “ageing in place”?

What do you consider a “safe” return home?

What are “home care services” for you?

What is a “home visit”?

What is the purpose of a “home visit”?

Case managers’ role

What are the main tasks of a case manager?

What is doing “case management”?

What is a “psychosocial follow-up”?

Assessing older adults receiving home care

What is an “assessment”?

How do you do an “assessment”?

What’s a “ISO-SMAF profile”?

Requesting home support services

What an “emergency home care allocation request”?

How do you define “quality home care services”?

Performance assessment

How do you define a “statistic”?

What can be counted as a “statistic”?

What cannot be counted as a “statistic”?

***Part B : main tasks***

Requesting home support services

What do you do when there are delays once home care services have been granted, but cannot be provided?

Preparing the transition to long-term care homes

What do you do when between the moment when you have sent the older adult’s file to the “long term care home committee” and the moment where they decide where the older adult will live?

Assistive devices loan

How are you involved in the process, once you have made the request to the occupational therapy department?

***Part C : meeting conclusion / other points not covered, as requested by the case managers***

Our meeting is coming to an end. Is there anything else you think I should know to better understand your work?

Is there anything you'd like to ask me?

Thank you for this meeting. If you have any ideas or questions about what we've talked about, you can write them down and we can discuss them again at a second meeting or by phone, at a time that's convenient for you.

Can I call you if I have further questions or need clarification?

# Interview grid – home care programs without observations

Based on my observations, informal discussions, and interviews with other case managers, I have identified several areas I'd like to explore with you today. Please note that there are no right or wrong answers to my questions. I simply want you to tell me what you know about these areas. This interview will be divided into three blocks.

***Part A****: questions about your daily work*

***Part B****: questions related to the main tasks you do*

***Part C****: meeting conclusion / other points not covered, as requested by you*.

Follow-up questions:

- Explain to me with examples of actions (or tasks) you need to perform?
- (Who) or (What document/regulation/policy/procedure), if any, would be likely to give me more information on [insert area discussed]?

***Part A : daily work***

Description of the work

Can you tell me about your work day yesterday?

When I say document, this can include voicemails, e-mails, software, guides, policies, etc. Can you tell me if you've used documents? If so, which ones?

***Part B : main tasks***

Requesting home support services

Describe the actions you do when you request home support services, from the moment you identify a need with the older adult to the moment where the care is delivered.

- What documents do you use?
- What are the services that you can request?

Describe the actions you do when you need to request home care services when there are emergencies.

Referring to other professionals

Describe the actions you do when you need to refer an older adult to another professional (e.g., nurse, occupational therapist, etc.).

Preparing the transition to long-term care homes

When is home care no longer an option?

Describe the actions you do when you need to “create a file for long-term care homes”, from the moment you identify that the older adult can no longer stay at home to the moment you “close” the older adult’s file.

Managing hospitalisations

Describe the actions you do when you learn that one older adult is in the hospital, from the moment the person is admitted to the time he or she is discharged.

- How do you learn about the admission?
- How do you learn about the discharge?
- What do you do once the older adult is back home?

Getting a new file

Describe the actions you do when an older adult's file gets added to your case load, from the moment where a request is made to the home care programme, to the moment where the file is assigned to you.

- What do you do once you get the file?

Assessing the workload

Describe the actions you do when your workload is being assessed.

- What do you do before you meet your team leader?
- What happens once your workload has been assessed?

Conducting the yearly assessment

Describe the actions involved when you need to conduct an annual assessment.

***Part C : meeting conclusion / other points not covered, as requested by the case managers***

Our meeting is coming to an end. Is there anything else you think I should know to better understand your work?

Is there anything you'd like to ask me?

Thank you for this meeting. If you have any ideas or questions about what we've talked about, you can write them down and we can discuss them again at a second meeting or by phone, at a time that's convenient for you.

Can I call you if I have further questions or need clarification?
